# Comparative efficacy of oral drugs for chronic radiation proctitis — a systematic review

**DOI:** 10.1186/s13643-023-02294-2

**Published:** 2023-08-22

**Authors:** Liangzhe Liu, Nana Xiao, Jinjun Liang

**Affiliations:** 1 Department of Clinical Pharmacy, School of Pharmacy, Guangdong Pharmaceutical University, Guangzhou, China; 2 Department of Colorectal Surgery, The Affiliated TCM Hospital of Guangzhou Medical University, Guangzhou, China; 3 Department of Surgery, Guangzhou University of Chinese Medical, Guangzhou, China; 4 School of Clinical Integrative Chinese and Western Medicine, Guangzhou Medical University, Guangzhou, China

**Keywords:** Chronic radiation proctitis, Oral treatments, Diarrhoea, Rectal bleeding, Radiation toxicity

## Abstract

**Background:**

Chronic radiation proctitis (CRP) is a long-term complication of pelvic radiotherapy that manifests as rectal bleeding, diarrhoea, fistula formation and obstruction. Treatments such as endoscopic argon plasma coagulation, hyperbaric oxygen therapy and rectal topical formalin have imposed a significant medical burden on CRP patients. In contrast, oral therapies offer a more accessible and acceptable option for managing CRP. Here, we conducted a systematic review of the efficacy of oral treatments for CRP to assess their potential as an effective and convenient treatment option for this condition.

**Methods:**

We searched the Cochrane Central Register of Controlled Trials, PubMed, Web of Science, China National Knowledge Infrastructure and Chinese VIP in February 2021. We included post-radiotherapy participants with CRP that compared oral medicine alone or in combination with other treatments versus control treatments. The primary outcomes were bleeding, diarrhoea and symptom score. Heterogeneity between studies was checked using Cochrane *Q* test statistics and *I*^2^ test statistics. The Cochrane risk-of-bias tool was used to assess the quality of the included studies.

**Results:**

We included 10 randomised controlled trials (RCTs) and 1 retrospective study with 898 participants. Three placebo-controlled trials evaluated the effects of oral sucralfate on CRP, with meta-analysis showing no significant different with placebo arm. Four trials on TCM demonstrated significant improvement of symptoms, especially for the 3 trials on oral TCM drinks. Retinyl palmitate and high-fibre diet were found to reduce rectal bleeding. The combination of oral pentoxifylline and tocopherol did not significantly change the process of CRP.

**Conclusions:**

Our study implies that oral TCM drinks, retinyl palmitate and a high-fiber diet showed significant improvement in CRP symptoms, but not with the combination of oral pentoxifylline and tocopherol. Further multicentre, larger-scale RCTs are needed to confirm the efficacy and safety of these treatments and optimize treatment strategies, ultimately improving the quality of life for patients with CRP.

**Supplementary Information:**

The online version contains supplementary material available at 10.1186/s13643-023-02294-2.

## Background

As the global cancer epidemic continues to rise and post-radiotherapy cancer prognosis improves, an increasing number of pelvic cancer survivors are presenting with chronic radiation proctitis (CRP) and seeking medical treatment [1, 2]. Significant bowel injury occurs in up to 6% of patients receiving pelvic irradiation [3, 4]. This long-term complication of pelvic radiotherapy occurs 3 months to years after pelvic radiotherapy, presenting with diarrhoea, rectal pain and/or haemorrhage in low grade, and 10% of the CRP may become high-grade, severe CRP presented with fistula formation or obstruction, having a substantial impact on patient’s quality of life [1]. And the cost of physical therapy and/or surgery has led to heavy medical burden for CRP patients.

Current standard treatment of CRP includes endoscopic therapies, nonsurgical treatments and surgeries. Nonsurgical treatment for CRP, as one of the late radiation tissue injury, composes of endoscopic argon plasma coagulation, radiofrequency ablation, hyperbaric oxygen therapy and rectal topical formalin, misoprostol or antibiotics [3, 5–7]. Oral antibiotics would be used only when systematic bacteraemia or sepsis was about to occur. Oral 5-aminosalicylic acid including olsalazine and mesalazine that are widely applied in chronic colitis has been contraindicated for pelvic radiation therapy. Recent studies began to focus on oral treatments such as for routine CRP treatments [8, 9].

Various medications and supplements have been studied for their potential in treating CRP, with different mechanisms of action and benefits. These include sucralfate, traditional Chinese medicine (TCM) drinks, probiotics, retinyl palmitate, a combination of vitamins C and E and pentoxifylline. Sucralfate binds to various epidermal growth factors, which can help reduce microvascular injury by stimulating angiogenesis. TCM for CRP typically contain multiple herbs, such as Sanguisorbae Radix, *Bletilla striata*, *Phellodendron* and Radix Paeoniae Rubra, which are associated with functions like detoxification, haemostasis and analgesia [10, 11]. TCM may also help regulate gut microbiota homeostasis and reduce chronic inflammation [12]. Probiotic supplements containing *Lactobacillus* spp. have been used to prevent or treat acute radiation proctitis-induced or 5-fluorouracil(5-FU)-based diarrhoea after pelvic cancer therapies [8, 13, 14]. Butyrate, a key metabolite of probiotics, serves as an energy source for colonocytes, and a deficiency in butyrate may lead to mucosal hyperplasia and acute or chronic inflammation [15, 16]. Retinyl palmitate has been demonstrated to promote wound healing by increasing cross-linking of collagen and myofibrils [17]. The combination of vitamins C and E, as antioxidants, reduces bleeding, diarrhoea and urgency [18]. Pentoxifylline, a phosphodiesterase inhibitor, serves as immunomodulator that down-regulates cytokines as well as a fibrogenic reaction mediator after irradiation which eases radiation-induced inflammatory and fibrotic process [19].

Oral treatments may provide advantages for CRP as it is claimed to be effective in improving CRP sign and symptom with more accessibility and lower medical burdens. However, the oral treatment options for CRP have not been clearly defined, and systematic review focusing on CRP oral drug is absent. Here, we sought out to assess the benefits and harms of oral treatments for CRP.

## Methods

### Search strategy and selection criteria

We followed Preferred Reporting Items for Systematic Reviews and Meta-Analyses (PRISMA) reporting standards for systematic reviews and meta-analyses [20]. All randomised controlled trials (RCTs) or historic/retrospective control group study in both English and Chinese, irrespective of publication status, which compared any oral intervention for CRP to no intervention, placebo or any other intervention was eligible for inclusion. Any person who had been treated with pelvic radiotherapy after more than 3 months, with or without chemotherapy, subsequently developed CRP of any grade. We accepted any oral treatment for CRP, including traditional Chinese medicine (TCM), sulfasalazine, glutamine, probiotics and vitamins in any dosage, and no intervention, placebo or any other nonsurgical intervention as control. We considered signs and symptoms scoring systems, including urgency, diarrhoea, rectal pain, hemorrhage, fistula formation and obstruction as primary outcomes. We determined the mortality, morbidity and quality of life (QoL) as secondary outcomes. Scoring systems of QoL involved Karnofsky performance status (KPS) as secondary outcomes.

In February 2021, we conducted a search for relevant studies in the Cochrane Central Register of Controlled Trials (CENTRAL), PubMed, Web of Science, China National Knowledge Infrastructure (CNKI) and Chinese VIP. No restrictions were placed on language or publication type (Additional files 1, 2, 3, 4, and 5 for the search strategies used for each database). In addition, we searched the Chinese Trial Registry (www.chictr.org) using the keywords “proctitis” or “proctopathy” and the prospective trial register (ClinicalTrials.gov) using the keywords (proctitis OR proctitides OR proctopathy OR proctocolitis OR proctosigmoiditis OR rectitis OR rectocolitis OR rectocolitides OR rectosigmoiditis) AND (radiation OR radiotherapy). Furthermore, we searched reference lists to identify unpublished trials, ongoing trials, confidential reports and raw data from published trials. We followed the instructions given in the *Cochrane Handbook for Systematic Reviews of Interventions* [21].

### Selection of studies and data management

One review author (L. Z. L.) was responsible for handsearching and identification of appropriate studies for consideration and entered all possibly relevant studies into a bibliographic software package Reference Manager (RefMan 5). Three review authors (L. Z. L., J. J. L. and N. N. X.) examined the electronic search results and independently reviewed the studies. Randomized controlled trials (RCTs) and retrospective studies that investigated the effectiveness of various oral medications and supplements for treating CRP were included. We retained studies when one or more review authors identified them as appropriate. We resolved any disagreements through discussion or, if required, by consulting a third review author (J. J. L.). We excluded trials that failed to meet our inclusion criteria, and the reasons are listed in the “Characteristics of excluded studies table” (supplementary document). The review authors all had content expertise in clinical practice, two had content expertise in medicine (L. Z. L. and N. N. X.) and one (J. J. L.) is an expertise in clinical surgery.

One review author (L. Z. L.) extracted relevant population and intervention characteristics using a standard data extraction template. Another author (J. J. L.) resolved any disagreements by discussion. We planned to solve all relevant missing information about the trials from the original authors of the articles. We resolved disagreements through discussion. Two review authors (L. Z. L., N. N. X.) independently assessed the risk of bias of all included studies according to the *Cochrane Handbook for Systematic Reviews of Interventions* [21]. We resolved disagreements through consensus. We allocated the level of evidence using the Grading of Recommendations Assessment, Development and Evaluation (GRADE) system [22]. RefMan 5 was used to assess the quality of evidence in accordance with selection bias, performance bias, detection bias, attrition bias and reporting bias. Evidence qualities were evaluated as high quality, medium quality, low quality or very low quality. The ROBINS-I tool was used to assess included non-randomized observational studies. Each domain was judged among the following options: low, moderate, serious, critical or no information. A final judgment with the same options was then made for the entire study based on the findings from each domain.

### Data analysis

We reported dichotomous data as risk ratios (RR) with 95% confidence intervals (CI), while for events with low probability, we utilized the Peto odds ratio (OR). Continuous variables were presented as mean differences (MD) with 95% CI. We assessed statistical heterogeneity using both the *χ*^2^ and *I*^2^ statistics, and clinical heterogeneity was evaluated in subgroup analysis. We considered *P* < 0.1 as evidence of statistical heterogeneity and an *I*^2^ value greater than 50% as indicative of significant statistical heterogeneity [21]. To account for expected clinical and methodological heterogeneity among the included trials, we used a random-effects model for meta-analysis of outcome measures from individual trials. The collected studies were categorized based on the type of oral medication or supplement being studied.

Our statistical analyses were conducted in accordance with the guidelines set out in the *Cochrane Handbook for Systematic Reviews of Interventions*. We summarized data that was sufficiently similar and of sufficient quality, with both event (dichotomous) data and continuous data. Dichotomous data was expressed as RR, while for low event rates, we utilized the Peto OR. Continuous data was presented as MD. When using RR or MD, we calculated overall results based on a fixed-effects model. Results from clinically comparable trials were reported separately.

## Results

### Characteristics of included studies

**Fig. 1:**
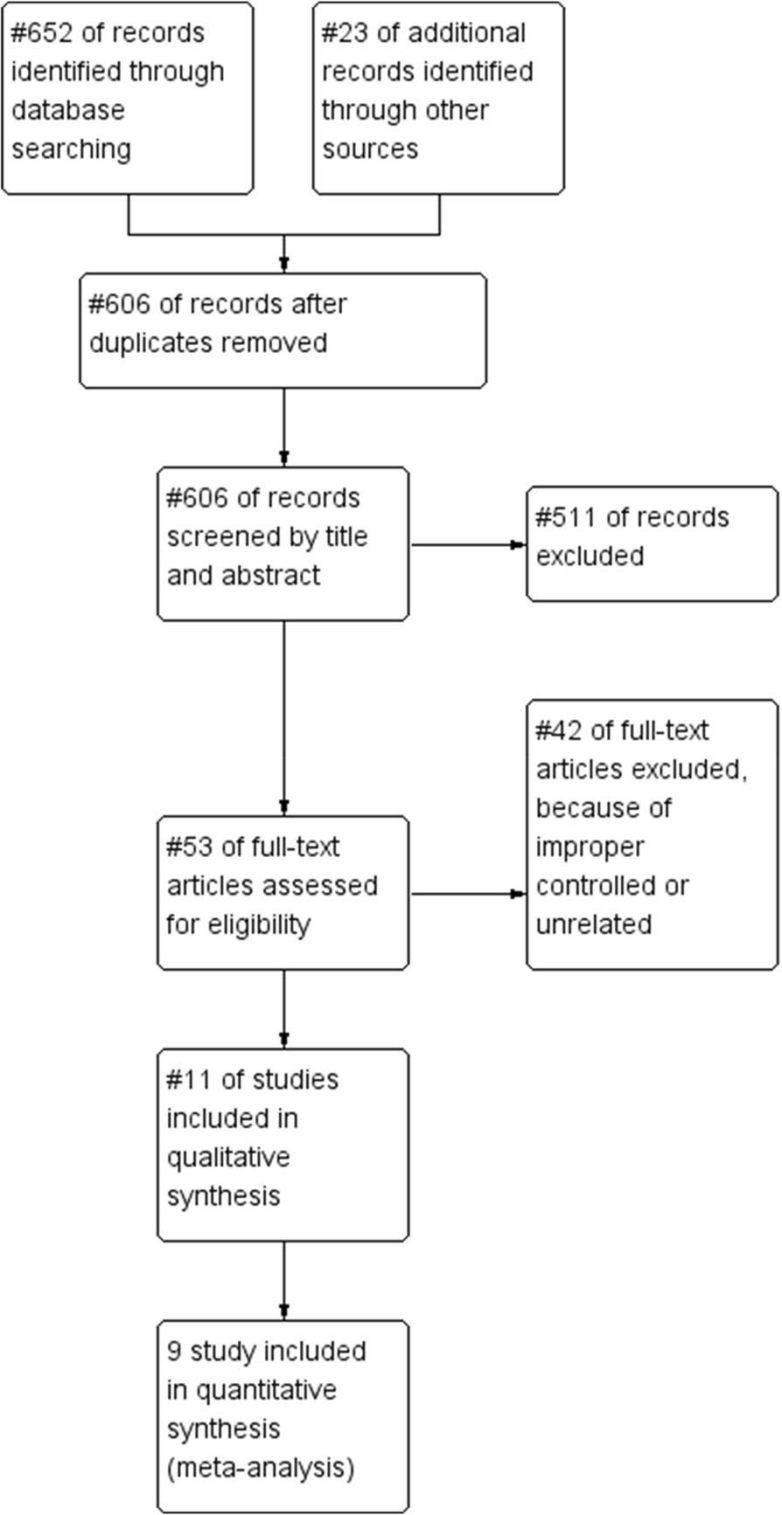
Study flow diagram

The bibliographic research generated a total of 675 references. After deduplication, 606 unique records were suitable for title and abstract screening. After title and abstract screening, 53 references remained for full-text screening. Ongoing studies and finished study without full-text report were not included. At the end of full-text review, a total of 11 references fully met our inclusion criteria and were considered eligible for data extraction. Ten randomised control trials (RCTs) and 1 retrospective study were included for qualitative and quantitative analysis (Fig. 1). No multicentre clinical trial was found. Four clinical control trials were conducted with placebo [17, 23–25]. Six reports were published in English and 5 published in Chinese. Four trials evaluated the effect of oral TCM [12, 26–28], 3 trials focused on the oral application of sucralfate [23–25] and a trial tested retinyl palmitate in radiation-induced intestinal inflammation [17]. Other studies on the application pentoxifylline [29], butyrate [19] and high-fibre diet [30] were also documented.

Eight-hundred ninety-eight patients in total were included in all 11 trials. Three studies included post-irradiation for various of disease including prostate, uterine, cervical, rectal or vaginal cancer [23, 24, 30]. Amoung these trails, some studies on Gynecological tumors included female specifically [27, 31], while some focus on prostate cancers which only occurs in male [25]. Four studies did not point out specific original cancer site [12, 17, 19, 29]. Detailed information of included studies and corresponding risk of bias was listed in Supplementary Table series S1.

### Risk of bias

Seven studies clearly indicated patients were randomly assigned [23–25, 27, 30, 31]. As for random sequence generation, the use of computer or the Doll’s randomization list was considered as low risk of selection bias. Hille’s study was retrospective and therefore categorized in high risks of selection bias. Five studies suggested that double blind was performed in their studies [17, 23–25, 31], which were scored in low risk of performance and detection bias. We cannot draw conclusion whether outcome data of Hille et al. were completed with confidence as they are retrospective studies. No missing data including drop-outs were documented by the end of the studies, and outcomes were clearly described in other trials. Report data were consistent with protocol provided by Chruscielewska et al., and this study is considered as low risk [23]. Other included studies presented available protocols, and we judged them as having an unclear risk of bias for this domain. We defined Hille, Christiansen, Pradier, Hermann, Siekmeyer and Weiss with moderate bias on confounding, no information in selection of participants, low bias on classification of interventions, deviations from the originally stated intervention, missing data, measurement of outcomes and selection of the reported result.

**Fig. 2:**
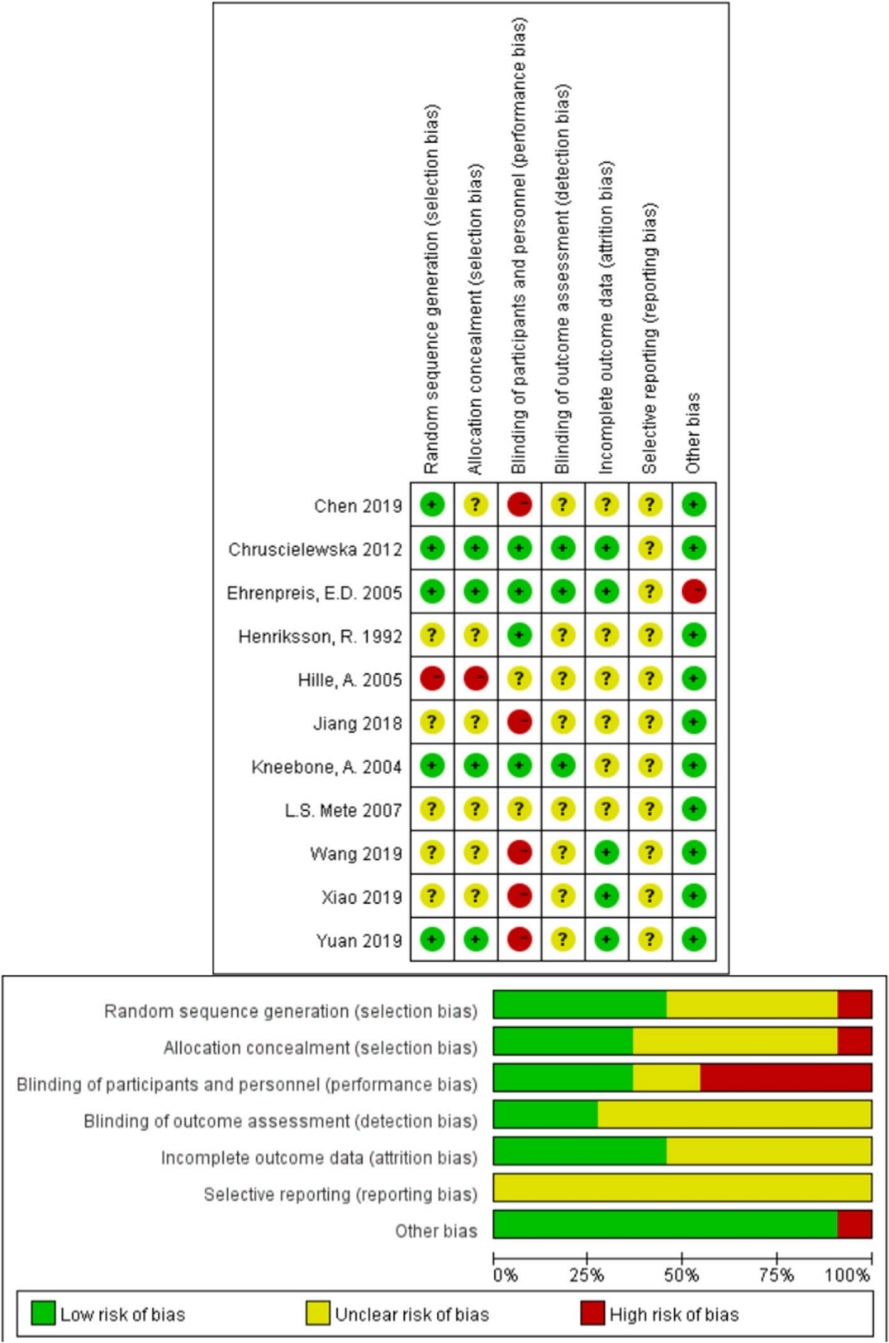
Risk-of-bias graph and summary: review authors’ judgements about each risk of bias item for each included study

Three studies involving oral application of Chinese medicine may be identical in terms of smell and taste, and blinding was not clearly announced in the report. We consider these papers as high risk of performance bias [12, 27, 30]. We cannot draw solid conclusion whether report of Hille et al. was complete [29]. Very small population (< 20 participants in total) did Ehrenpreis include in their studies, and we cannot exclude the possible systematic bias. We did not identify any other potential bias, and we rated this domain at low risk of bias (Fig. 2).

Overall, 3 randomised, double-blinded, placebo-controlled trials on the effect of sucralfate; 4 randomized controlled trials on TCM; 3 trials on daily supplements including vitamin A, butyrate and high-fibre diet; and 1 trial which combined pentoxifylline and tocopherol were analysis. Most of the trials made use of comprehensive scoring system for evaluation and data presentation, such as RTOG/EORTC toxicity grades [25, 27, 29], KPS score [27, 31] and Vienna rectoscopic score [27]. Some studies applied self-developed scaling systems [17, 19, 24, 31]. These scaling systems commonly involve diarrhoea frequency, bleeding, pain and surgery possibility. And we grouped specific outcomes dichotomously by the conclusive determination from each article.

### Effects of interventions

**Fig. 3:**
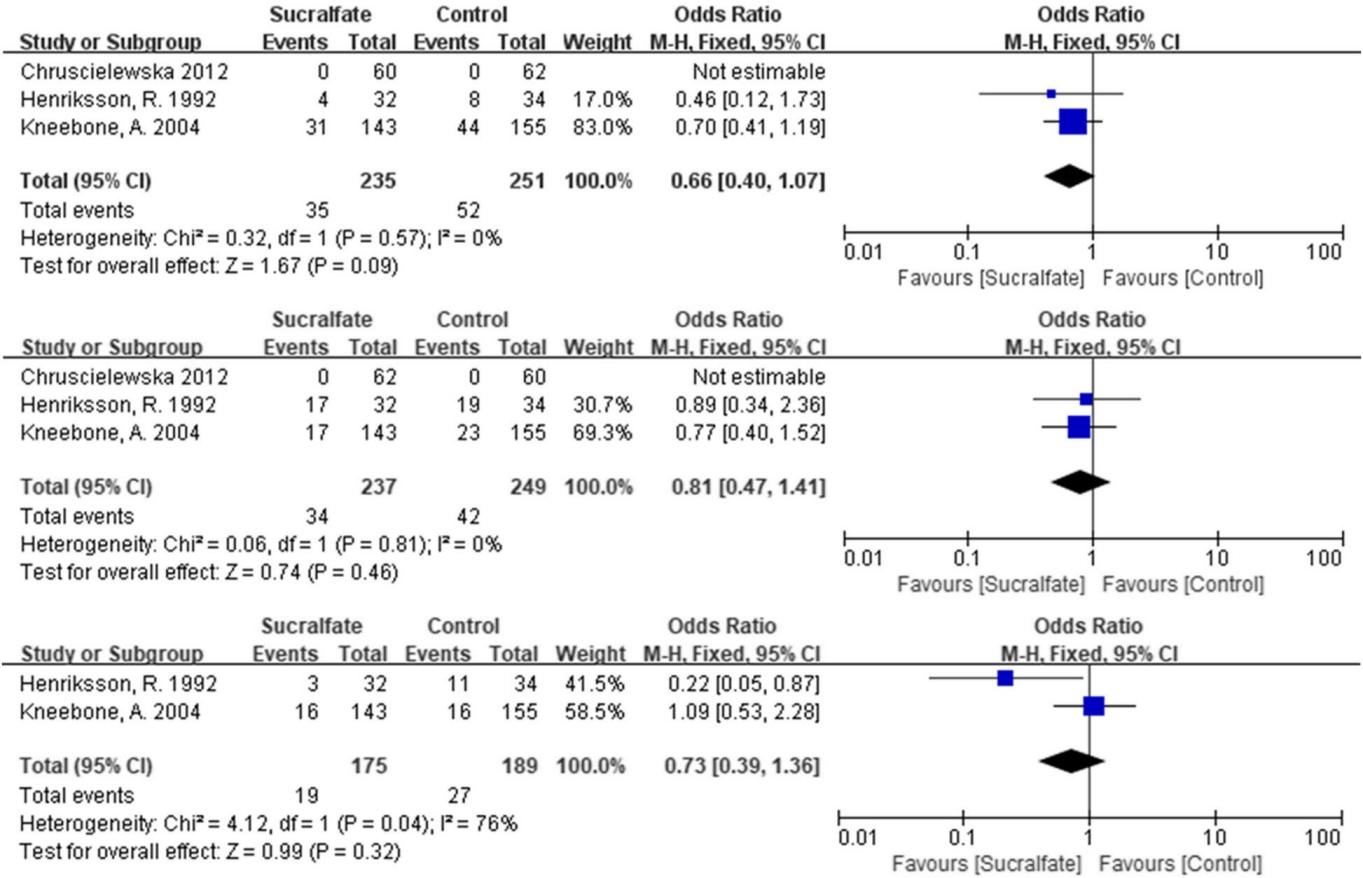
Forest plots showing the study-specific estimates of the odds ratios as compared to corresponding control populations with placebo, by overall diarrhea score, bleeding score or the presence of high stool frequency

Three randomised, double-blinded, placebo-controlled trials on the effect of sucralfate have been reported [23–25]. In total, 235 and 251 participants were included throughout the three studies in sucralfate arm and placebo arm, respectively. Outcome documented in the three studies were commonly based on the overall score of clinical symptoms involving diarrhoea score and bleeding score. Low heterogeneity was found in the three studies in terms of diarrhoea score (*χ*^2^ = 0.32, *df* = 1, *P* = 0.57; *I*^2^ = 0%) or bleeding score (*χ*^2^ = 0.06, *df* = 1, *P* = 0.81; *I*^2^ = 0%). No significant difference between the effect of sucralfate or placebo was found in the three studies regarding diarrhoea (*OR* = 0.81, 95% *CI* = [0.47, 1.41]) or bleeding (*OR* = 0.81, 95% *CI* = [0.47, 1.41]). One RCT with 198 participants weighted 83% of the meta-analysis, reported each of the RTOG toxicity grades, number of worse bleeding and frequency and showed no significant difference between two arms in all aspects. Chruscielewska et al. showed no change in diarrhoea score or bleeding score from subacute phase (week 8) to chronic phase (week 52, Fig. 3, Table 1).

**Table 1:** Summary of data analysis

Outcome or subgroup	Studies	Participants	Statistical method	Effect estimate
1. Sucralfate				
1.1 Overall diarrhoea score	3	486	Odds ratio (M-H, fixed, 95% CI)	0.66 (0.40, 1.07)
1.2 Bleeding	3	486	Odds ratio (M-H, fixed, 95% CI)	0.81 (0.47, 1.41)
1.3 High frequency	2	364	Odds ratio (M-H, fixed, 95% CI)	0.73 (0.39, 1.36)
2 Chinese medicine				
2.1 High symptom score	4	232	Odds ratio (M-H, fixed, 95% CI)	0.18 (0.10, 0.34)
2.2 High KPS score	1	55	Mean difference (IV, random, 95% CI)	0.24 (−3.89, 4.37)
2.3 Haemoglobin	1	55	Mean difference (IV, fixed, 95% CI)	4.82 (−2.14, 11.78)
3 Dietary and supplementary treatments				
3.1 Reduced bleeding	2	80	Mean difference (IV, random, 95% CI)	6.02 (1.75, 10.30)
3.2 Endoscopic evaluation	1	70	Odds ratio (M-H, fixed, 95% CI)	3.89 (1.34, 11.25)
3.3 BMI	1	63	Mean difference (IV, fixed, 95% CI)	−0.15 (−1.04, 0.74)
4. Pentoxifylline and tocopherol				
4.1 Relieved symptom	1	30	Odds ratio (M-H, fixed, 95% CI)	5.00 (0.93, 26.79)

**Fig. 4:**
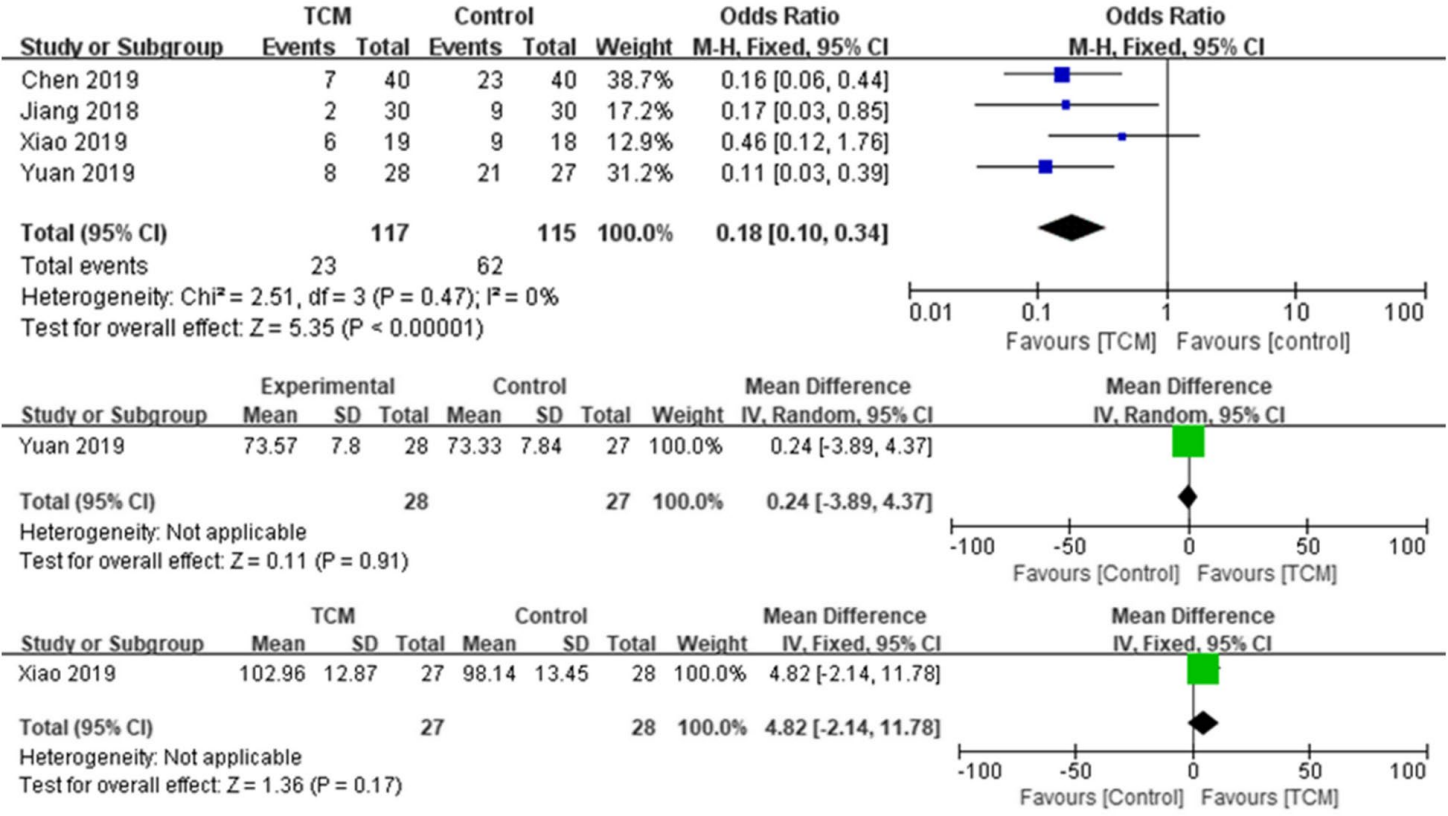
Forest plots showing the study-specific estimates of the odds ratios for effect of TCM (experimental) in the presence of high-symptom score, or mean difference for KPS score or hemoglubin, as compared to corresponding control populations

Four randomised controlled trials were conducted to evaluate the effect of TCM on totally 232 participants [12, 26, 27, 31]. The four trials did not show high heterogeneity (*χ*^2^ = 2.52, *df* = 4, *P* = 0.64; *I*^2^ = 0%). Meta-analysis result shows that TCM is in favour for reducing symptom of CRP (*OR* = 0.18, 95% *CI* = [0.10, 0.34]; *Z* = 5.35, *P* < 0.00001, Fig. 4).

Combination of oral TCM therapies and probiotics was applied in these studies. In Chen et al., oral treatment and enema with dexamethasone, gentamicin and lidocaine were compared with *Entrocoordinatibiogen*, a *Bacillus licheniformis* capsule; the addition of TCM showed advantages in reducing diarrhoea, hematochezia and serum TNF-α, IL-6, IL-8 and IL-10. Oral smectite was used by Xiao et al. The combination of bifid triple viable, probiotics composed of enterococcus, *Lactobacillus acidophilus* and *Bifidobacterium* improved symptoms of CRP but did not significantly altered quality of life (*OR* = 0.24, 95% *CI* = [−3.89, 4.37]).

**Fig. 5:**
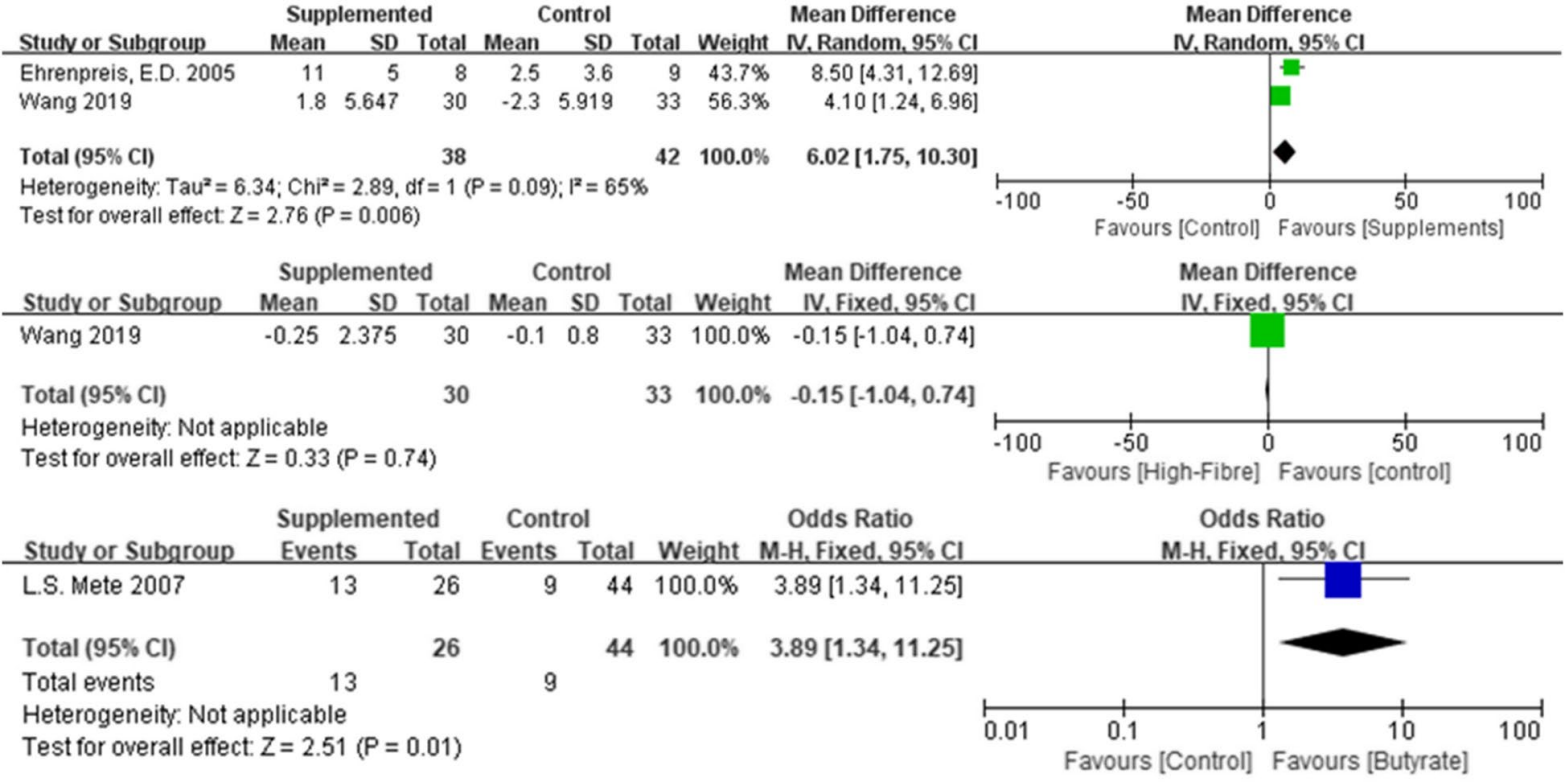
Forest plot showing the study-specific estimates of the mean difference for daily supplements on reducing rectal bleeding score or BMI, or odds ratio on endoscopic finding, as compared to corresponding control populations

We classified the application of retinyl palmitate [17], butyrate [19] and high-fibre diet (HFD, [30]) as supplements and compared their effect on radiation proctitis using meta-analysis. Ehrenpreis and Wang’s studies are heterogeneity, and we used random effect model instead for evaluation (Tau^2^ = 6.34; *χ*^2^ = 2.89, *df* = 1, *P* = 0.09; *I*^2^ = 65%). The supplementary of both VitA and high-fibre diet resulted in reduction of bleeding and increase in haemoglobin (*Z* = 2.76, *P* = 0.006, *MD* = 6.02, 95% *CI* = [1.75, 10.30], Fig. 5). Effect of VitA was estimated by the scale developed by the author, the Radiation Proctopathy System Assessments Scale (RPSAS). And Wang demonstrated that the effects of HFD on CRP, including change in haemoglobin level and inflammation factors level, were not relying on the change of BMI (*P* = 0.74). It should be noted that HFD was claimed prescribed before and during radiotherapy, but Wang did not clearly indicate the time line of data collection. The improvement mediated by HFD may be transient, but the authorship did not precisely recommend the duration of HFD for CRP. Mete, Assisi and Casale conducted endoscopic for 70 participants, and those who received butyrate showed better condition under endoscope (*Z* = 2.51, *P* = 0.01), in terms of telangiectasia, adjacent mucosa, ulcers, stenosis or necrosis. Since the data symptoms were significantly different at T0 (before treatment), we did not include this study in meta-analysis.

**Fig. 6:**
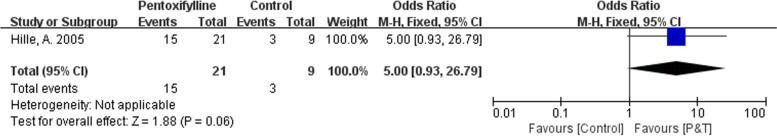
Forest plot showing the study-specific estimates of the odds ratios for the combination of pentoxifylline and tocopherol (Pentoxifylline) as compared to corresponding control populations

We consider the combination therapy of pentoxifylline and tocopherol (PT) as a separated treatment. Despite tocopherol (VitE) serves as a daily supplementary, pentoxifylline is a prescription which is not over the counter. The effect of PT was evaluated by RTOG/EORTC toxicity criteria, and the PT arm demonstrated no worsening, but the improvement was not statically significant (*OR* = 5.00, 95% *CI* = [0.93, 26.79], Fig. 6). It should be noted that 6 patients who were treated with pentoxifylline and tocopherol received additional symptomatic therapy with smectite (*n* = 1), laxatives (*n* = 1), short-chain fatty acids (*n* = 3) or misoprostol (*n* = 1). The improvement in RTOG grading may not be solely result from PT treatment [29].

## Discussion

This systematic review and meta-analysis of 11 studies found that oral TCM drinks, but not sucralfate, demonstrated significant symptom improvement in CRP. In addition, probiotics and retinyl palmitate may have potential benefits in reducing inflammation and improving symptoms. To our knowledge, this is the first systematic review summarizing oral treatment for CRP. Oral TCM seems to be useful for increasing haemoglobin, reducing CRP symptom and improving overall quality of life. But the complexity nature of herbs and the combination of several herbs together with the use of smectite and other treatment raise the concern of heterogeneity in general application. VitA and high-fibre diet remitted rectal bleeding, but few participants (17 and 63 participants) were included.

The present study is in line with previous findings. Wetering et al. reviewed a series of nonsurgical interventions for late rectal problems after pelvic radiotherapy including enema, hyperbaric oxygen therapy and argon plasma coagulation. Updated version in 2016 included some of outcome data of sucralfate trials without meta-analysis, and 1 TCM trial consists of Shen Ling Bai Zhu powders administered through anorectal [7]. Zhou et. al systematically compared the therapeutic effect with and without combination of TCM on acute radiation proctitis which also showed significant benefit [32].

TCM seems to be useful to reduce rectal bleeding without severe side effect on the liver or kidney [12, 33]. But the dosage and herbs composition variated between trials, decreasing the confidence of meta-analysis and the applicability worldwide. Well-designed, large-scale, multicentre placebo-controlled trial should be conducted in the future for validation. Neither 6 g/day nor 12 g/day sucralfate may be efficient to improve symptom of CRP. The application of daily supplements may relieve CRP, but more evidence should be provided. The combination of 800-mg pentoxifylline and 1000-mg tocopherol daily did not significantly improve CRP symptom. The sole effect of pentoxifylline or tocopherol remains unclear [29, 34–36].

This evidence implies that TCM, probiotics and prebiotics are potential treatment options for CRP. Coptidis, *Glycyrrhiza* and *Pulsatilla* were commonly used in TCM treatment, and the active ingredients of these herbs may be found to promote a uniform prescription. Animal studies have shown that an array of TCM treatments may protect colorectal tissue from fibrosis, apoptosis or inflammation, indicating the potential for future clinical application. Probiotics have also been applied to improve symptoms of bowel diseases such as inflammatory bowel disease (IBD). Although the effect of butyrate as a metabolite of probiotics was not statistically significant, probiotics have been demonstrated to relieve diarrhoea in acute radiation proctitis. Vitamin A, vitamin E and butyrate have also been supplemented for CRP treatment, and a double-blind placebo-controlled trial is needed to confirm the benefit. Finally, a specific scaling system should be developed to uniformly assess the severity of CRP symptoms, including diarrhoea, rectal bleeding, ulcers and quality of life.

Variated, comprehensive outcomes scoring systems in these trials make it difficult to be interpretated. Radiation Therapy Oncology Group toxicity scale [37–39], Karnofsky Performance Scale [40, 41], rectoscopic score and self-developed score were found to be used. A specific scaling system shall be developed to uniform the outcome assessment for CRP documenting the severity of diarrhoea, rectal bleeding, ulcer and the quality of life. The meta-analysis of sucralfate was based on Chutkan and Gilinski scale, RTOG toxicity sore and self-developed diarrhoea score.

Future trial is needed to test the contribution of novel oral therapies on CRP, such as probiotics, protein supplements and targeted medicine. Probiotics have been applied for improving symptoms of bowel diseases such as inflammatory bowel disease (IBD), Although effect of butyrate as a metabolite of probiotic was not statistically significant, probiotics have been demonstrated to relief acute diarrhoea. Delia et al. investigated the efficacy of a high-potency probiotic preparation on prevention of radiation-induced diarrhoea in 490 patients [13]. The combination of *Bifidobacterium longum*, *Lactobacillus acidophilus* and *Enterococcus faecalis* was also utilized by Yuan et al. in CRP [31]. But RCT on probiotics for CRP was not identified in this systematic review. Enteral and parenteral nutrition have been widely applied for an array of IBD and protein/amino acids supplementary treatment for cancer [42, 43]. More evidence is expected to investigate the effect of CRP-induced malnutrition correction on adjusting anaemia and enhancing recovery. Recent study on molecular mechanism of CRP implied that platelet-derived growth factor C signaling is a potential therapeutic target in animal model [44]. Targeted medicine may be developed as another promising oral treatment for CRP.

## Conclusion

Pelvic cancer survivors are suffering from CRP, presenting with prolong diarrhoea, rectal bleeding, ulcers or even fistula, which requires surgery. Oral drugs are convenience for daily application, and some have been reported improved symptoms of CRP. However, the treatment strategy of CRP remains controversial. We systematically reviewed the efficacy of oral treatment for CRP and found that TCM, daily supplement with high-fibre diet and vitamin A contributed to reduce rectal bleeding and diarrhoea. In spite of oral sucralfate, current oral treatments should be suggested for relieving symptom of CRP. The effect of enteral nutrition, probiotics, smectite and other commonly used oral CRP treatment may be evaluated in future.

### Supplementary Information

**Additional file 1.** 

**Additional file 2.** 

**Additional file 3.** 

**Additional file 4.** 

**Additional file 5.** 

**Additional file 6.**

## Data Availability

Not applicable. This is a systematic review of published literature.

## Abbreviations

CRP: Chronic radiation proctitis
RCTs: Randomized control trials
CI: Confidence intervals
OR: Odds ratio
MD: Mean difference
HFD: High-fibre diet
TCM: Traditional Chinese medicine
GRADE: Grading of Recommendations Assessment, Development and Evaluation
