# Supplementary material for: Comparative efficacy of oral drugs for chronic radiation proctitis — a systematic review
Source: Syst Rev. 2023 Aug 22;12:146. doi: 10.1186/s13643-023-02294-2 (PMC10464232; doi:10.1186/s13643-023-02294-2)
Supplement: Supplementary file 1 — Additional file 1. [file 13643_2023_2294_MOESM1_ESM.docx]

Appendix 1 Search Strategy for CENTRAL

ID Search Hits

#1 MeSH descriptor: [Proctitis] explode all trees 140

#2 (proctitis or proctitides or proctopathy or proctocolitis or proctosigmoiditis or rectitis or rectocolitis or rectocolitides or rectosigmoiditis) 669

#3 (rect* or anus or anal or anorectal) near/5 (injur* or inflam* or diseas* or bleed* or rupture* or discharge* or pain* or discomfort* or irritat*) 4843

#4 #1 OR #2 OR #3 5334

#5 MeSH descriptor: [Radiotherapy] explode all trees 6131

#6 MeSH descriptor: [] explode all trees and with qualifier(s): [radiotherapy - RT] 8218

#7 radiotherap* or radiat* or irradiat* or radiochemo* or chemoradio* 54960

#8 #6 OR #5 OR #7 55037

#9 #4 AND #8 864

#10 CHRONIC OR LATE 176896

#11 #9 AND #10 in Cochrane Protocols, Trials, Clinical Answers, Editorials, Special Collections 236

#12 (oral) 211829

#13 #12 and #11 15
