# Supplementary material for: Comparative efficacy of oral drugs for chronic radiation proctitis — a systematic review
Source: Syst Rev. 2023 Aug 22;12:146. doi: 10.1186/s13643-023-02294-2 (PMC10464232; doi:10.1186/s13643-023-02294-2)
Supplement: Supplementary file 2 — Additional file 2. [file 13643_2023_2294_MOESM2_ESM.docx]

Appendix 2 Search Strategy for Pubmed

(((proctitis OR proctitides OR proctopathy OR proctocolitis OR proctosigmoiditis OR rectitis OR rectocolitis OR rectocolitides OR rectosigmoiditis) OR (proctitis[MeSH Terms]) OR (rect* OR anus OR anal OR anorectal)) AND ((radiotherapy[MeSH Terms]) OR (radiotherap* or radiat* or irradiat* or radiochemo* or chemoradio*)) AND (chronic OR late)) AND (oral)
