# Supplementary material for: Comparative efficacy of oral drugs for chronic radiation proctitis — a systematic review
Source: Syst Rev. 2023 Aug 22;12:146. doi: 10.1186/s13643-023-02294-2 (PMC10464232; doi:10.1186/s13643-023-02294-2)
Supplement: Supplementary file 3 — Additional file 3. [file 13643_2023_2294_MOESM3_ESM.docx]

Appendix 3 Search Strategy for CNKI

FT = (proctitis OR proctitides OR proctopathy OR proctocolitis OR proctosigmoiditis OR rectitis OR rectocolitis OR rectocolitides OR rectosigmoiditis) AND FT = (radiotherapy or radiation or irradiation or radiochemotherapy or chemoradiotherapy) AND FT = (chronic OR late) AND FT = (oral)
