# Supplementary material for: Comparative efficacy of oral drugs for chronic radiation proctitis — a systematic review
Source: Syst Rev. 2023 Aug 22;12:146. doi: 10.1186/s13643-023-02294-2 (PMC10464232; doi:10.1186/s13643-023-02294-2)
Supplement: Supplementary file 4 — Additional file 4. [file 13643_2023_2294_MOESM4_ESM.docx]

Appendix 4 Search Strategy for Chinese VIP

U=(proctitis OR proctitides OR proctopathy OR proctocolitis OR proctosigmoiditis OR rectitis OR rectocolitis OR rectocolitides OR rectosigmoiditis) AND U=(radiotherapy OR radiation OR irradiation OR Radiochemotherapy OR chemoradiotherapy) AND U=(chronic OR late)
