# Supplementary material for: Comparative efficacy of oral drugs for chronic radiation proctitis — a systematic review
Source: Syst Rev. 2023 Aug 22;12:146. doi: 10.1186/s13643-023-02294-2 (PMC10464232; doi:10.1186/s13643-023-02294-2)
Supplement: Supplementary file 5 — Additional file 5. [file 13643_2023_2294_MOESM5_ESM.docx]

Appendix 5 Search Strategy for Web of Science

ALL = (proctitis OR proctitides OR proctopathy OR proctocolitis OR proctosigmoiditis OR rectitis OR rectocolitis OR rectocolitides OR rectosigmoiditis) AND ALL = (radiotherap* or radiat* or irradiat* or radiochemo* or chemoradio*) AND ALL = (chronic OR late) AND ALL=(oral)
