# Supplementary material for: Comparative efficacy of oral drugs for chronic radiation proctitis — a systematic review
Source: Syst Rev. 2023 Aug 22;12:146. doi: 10.1186/s13643-023-02294-2 (PMC10464232; doi:10.1186/s13643-023-02294-2)
Supplement: Supplementary file 6 — Additional file 6. [file 13643_2023_2294_MOESM6_ESM.docx]

**Supplementary tables**

## Table series S1. Characteristics of included studies

### *Chen 2019*

| **Methods** | Randomized control trial |
| --- | --- |
| **Participants** | Eighty patients with radioactive proctitis who met the inclusion criteria |
| **Interventions** | The control group was treated with western medicine combined with enema, and the observation group was treated with oral spleen and clearing spleen combined with traditional Chinese medicine enema. |
| **Outcomes** | Symptoms were scored before and after treatment from abnormal sensation of bowel movement, blood in stool, stool traits and frequency, abdominal pain; TNF-α, IL-8, IL-6, IL-10 levels were detected by ELISA. The intestinal mucosal healing and recurrence were observed by microscopy. |
| **Notes** |  |

#### Risk of bias table

| **Bias** | **Authors' judgement** | **Support for judgement** |
| --- | --- | --- |
| Random sequence generation (selection bias) | Low risk | “Random number table” |
| Allocation concealment (selection bias) | Unclear risk | Allocation concealment was not mentioned |
| Blinding of participants and personnel (performance bias) | High risk | Blinding was not mentioned and the application of TCM and capsule may be identical |
| Blinding of outcome assessment (detection bias) | Unclear risk | Blinding was not mentioned |
| Incomplete outcome data (attrition bias) | Unclear risk | Dropout was not mentioned |
| Selective reporting (reporting bias) | Unclear risk | No protocol |
| Other bias | Low risk |  |

### *Chruscielewska 2012*

| **Methods** | Single-centre, randomised, placebo-controlled, double-blind study |
| --- | --- |
| **Participants** | Patients with haemorrhagic chronic radiation proctitis after irradiation for prostate, uterine, cervical, rectal or vaginal cancer. |
| **Interventions** | All patients received APC, and were then randomised to oral sucralfate (6 g twice a day) or placebo treatment for 4 weeks. APC was repeated every 8 weeks if necessary after the first session. |
| **Outcomes** | Patients were graded clinically and endoscopically according to the Chutkan and Gilinski scales before and at 8 and 16 weeks after initial APC treatment (1.5–2 l/min, 25–40 W) and after 52 weeks (clinical only) |
| **Notes** |  |

#### Risk of bias table

| **Bias** | **Authors' judgement** | **Support for judgement** |
| --- | --- | --- |
| Random sequence generation (selection bias) | Low risk | "computer generated allocation code" |
| Allocation concealment (selection bias) | Low risk | “The study investigators were not involved in the preparation of the computer-generated allocation code or in the generation of consecutively numbered containers with the study medication (drug or identical-appearing placebo tablets)” |
| Blinding of participants and personnel (performance bias) | Low risk | “Study participants and investigators remained blinded to group assignment until the conclusion of the study, which was the visit at week 52.” |
| Blinding of outcome assessment (detection bias) | Low risk | “The study investigators were not involved in the preparation of the computer-generated allocation code or in the generation of consecutively numbered containers with the study medication (drug or identical-appearing placebo tablets)” |
| Incomplete outcome data (attrition bias) | Low risk | “To compensate for nonevaluable patients and dropouts, an enrolment of 60 patients per arm was planned.” |
| Selective reporting (reporting bias) | Unclear risk | no protocol |
| Other bias | Low risk |  |

### *Ehrenpreis, E.D. 2005*

| **Methods** | Randomised, double-blind trial |
| --- | --- |
| **Participants** | Patients had significant symptoms after 6 months radiotherapy as measured with the Radiation Proctopathy System Assessments Scale. |
| **Interventions** | Oral administration of retinol palmitate (10,000 IU by mouth for 90 days) or placebo |
| **Outcomes** | The Radiation Proctopathy System Assessments Scale scores before and every 30 days for 90 days were measured.  Response was defined as a reduction in two or more symptoms by at least two Radiation Proctopathy System Assessments Scale points. |
| **Notes** | Few (19) participants may cause systematic bias |

#### Risk of bias table

| **Bias** | **Authors' judgement** | **Support for judgement** |
| --- | --- | --- |
| Random sequence generation (selection bias) | Low risk | "The pharmacy developed a random number system for treatment assignment." |
| Allocation concealment (selection bias) | Low risk | "The pharmacy developed a random number system for treatment assignment." |
| Blinding of participants and personnel (performance bias) | Low risk | "Retinol palmitate 10,000 IU (Nature’s Bounty Inc., Bohemia, NY) and identical placebo capsules were placed in containers of 100 capsules each by the Investigational Pharmacy at the University of Chicago.""Neither the investigators nor patients were aware who was receiving retinol palmitate or placebo." |
| Blinding of outcome assessment (detection bias) | Low risk | "Retinol palmitate 10,000 IU (Nature’s Bounty Inc., Bohemia, NY) and identical placebo capsules were placed in containers of 100 capsules each by the Investigational Pharmacy at the University of Chicago.""Neither the investigators nor patients were aware who was receiving retinol palmitate or placebo." |
| Incomplete outcome data (attrition bias) | Low risk | "The study was concluded in patients who responded to placebo or did not respond to retinol palmitate. " |
| Selective reporting (reporting bias) | Unclear risk | no protocol |
| Other bias | High risk | Systematic bias deal to small sample size |

### *Henriksson, R. 1992*

| **Methods** | Double‐blind randomised placebo‐controlled study; |
| --- | --- |
| **Participants** | 70 patients treated with radiotherapy of localized malignancies in the pelvis. Radiotherapy was delivered in a conventional manner with high‐energy photons in a total dose of 62‐66 Gy (target dose, 1.8‐2.2 Gy) during 6.5 weeks. |
| **Interventions** | Dose granules of sucralfate or placebo were given 2 weeks after irradiation started and continued for 6 weeks |
| **Outcomes** | Less problems with acute (5 weeks) and chronic (66 weeks) bowel discomfort.  The reduction of consumption of loperamide  Adverse effects |
| **Notes** | Some of the participants were having acute RP. |

#### Risk of bias table

| **Bias** | **Authors' judgement** | **Support for judgement** |
| --- | --- | --- |
| Random sequence generation (selection bias) | Unclear risk | Unknown |
| Allocation concealment (selection bias) | Unclear risk | Unknown |
| Blinding of participants and personnel (performance bias) | Low risk | "Dose granules of sucralfate and placebo identical in taste, colour, and consistency were dispensed randomly to each patient  2 weeks after radiotherapy started, with the instruction to ingest one dose package (1 g) dissolved in water six times daily" |
| Blinding of outcome assessment (detection bias) | Unclear risk | "Patients were interviewed by the same physician once every week and at the end of treatment. Two months and 66 weeks after termination of the radiotherapy the patients were interviewed again by the physician. The details of the bowel action registered  on the calendar were consecutively converted by the patients and the physician to a diarrhoea score during the period of investigation" |
| Incomplete outcome data (attrition bias) | Unclear risk | Unknown |
| Selective reporting (reporting bias) | Unclear risk | no protocol |
| Other bias | Low risk |  |

### *Hille, A. 2005*

| **Methods** | Retrospective analysis |
| --- | --- |
| **Participants** | 30 patients with radiation-induced proctitis/enteritis grade I-II according to the RTOG/EORTC toxicity criteria, |
| **Interventions** | 21 were treated with pentoxifylline and tocopherol. Depending on physician's decision nine patients received symptomatic treatment only. |
| **Outcomes** | Relief of symptoms  A reduction from grade I/II to grade 0 toxicity and from grade II to grade I toxicity  The median time to improvement  Deterioration of symptoms  No amelioration  Grade I toxicity with a spontaneous relief of their symptoms |
| **Notes** | SE: Three patients developed a coagulopathy, probably due to pentoxifylline. One patient with amelioration of proctitis symptoms from grade II to I suffered from rectal bleeding again 3 months after initiation of treatment. Rectal bleeding stopped after taking off pentoxifylline |

#### Risk of bias table

| **Bias** | **Authors' judgement** | **Support for judgement** |
| --- | --- | --- |
| Random sequence generation (selection bias) | High risk | Retrospective analysis |
| Allocation concealment (selection bias) | High risk | Retrospective analysis |
| Blinding of participants and personnel (performance bias) | Unclear risk | Retrospective analysis |
| Blinding of outcome assessment (detection bias) | Unclear risk | Retrospective analysis |
| Incomplete outcome data (attrition bias) | Unclear risk | "Depending on physician's decision, nine of 30 patients with chronic disease did not undergo a treatment with pentoxifylline and tocopherol." |
| Selective reporting (reporting bias) | Unclear risk | No protocol |
| Other bias | Low risk |  |

### *Jiang 2018*

| **Methods** | Randomized control trail |
| --- | --- |
| **Participants** | Patients with radiation enteritis |
| **Interventions** | The experimental group was given Hongyudecoction and the control group was given orfloxacin combine with SASP |
| **Outcomes** | Total effective grade and symptom score before and after treatment |
| **Notes** |  |

#### Risk of bias table

| **Bias** | **Authors' judgement** | **Support for judgement** |
| --- | --- | --- |
| Random sequence generation (selection bias) | Unclear risk | "randomly assigned" |
| Allocation concealment (selection bias) | Unclear risk | "randomly assigned" |
| Blinding of participants and personnel (performance bias) | High risk | Blinding was not mentioned and the taste and smell TCM may be identical |
| Blinding of outcome assessment (detection bias) | Unclear risk | Blinding was not mentioned |
| Incomplete outcome data (attrition bias) | Unclear risk | Dropout was not mentioned |
| Selective reporting (reporting bias) | Unclear risk | no protocol |
| Other bias | Low risk |  |

### *Kneebone, A. 2004*

| **Methods** | Multi-centre double‐blind, placebo‐controlled, randomised trial |
| --- | --- |
| **Participants** | Patients receiving definitive radiotherapy for prostate cancer |
| **Interventions** | Either 3 g of oral sucralfate suspension or placebo twice daily |
| **Outcomes** | The cumulative incidence of Radiation Therapy Oncology Group Grade 2 or worse late rectal toxicity at 2 years  Significant bleeding (Grade 2 or worse)  Bowel frequency  Mucus discharge  Fecal incontinence  Sigmoidoscopy findings, Grade 2 or worse rectal changes |
| **Notes** |  |

#### Risk of bias table

| **Bias** | **Authors' judgement** | **Support for judgement** |
| --- | --- | --- |
| Random sequence generation (selection bias) | Low risk | "Randomization was by a confidential computer generated list of patient trial numbers that was generated before the start of the trial." |
| Allocation concealment (selection bias) | Low risk | "The method of randomizations was a one-to-one stratified allocation within blocks of 8." |
| Blinding of participants and personnel (performance bias) | Low risk | "The trial patients, investigators, data managers, and pharmacy staff were kept unaware of the allotted treatment" |
| Blinding of outcome assessment (detection bias) | Low risk | "The trial patients, investigators, data managers, and pharmacy staff were kept unaware of the allotted treatment" |
| Incomplete outcome data (attrition bias) | Unclear risk | Dropout was not mentioned |
| Selective reporting (reporting bias) | Unclear risk | no protocol |
| Other bias | Low risk |  |

### *L.S. Mete 2007*

| **Methods** | Clinical control trail |
| --- | --- |
| **Participants** | Patients after radiotherapy |
| **Interventions** | 26 patients were included in the study and treated with oral butyrate in combination with topical Mesalazine and/or beclomethasone for 6 months and 44 control subjects received topical therapy with Mesalazine and/or beclomethasone for 6 months. |
| **Outcomes** | Assessment of clinical status was performed using the subjective and objective parameters of the LENT/SOMA scale: frequency of stools, tenesmus, pain, mucosal loss, diarrhoea and bleeding.  Based on the endoscopic scoring system given above, self-defined a new simplified classification that uses only the most common endoscopic parameters (telangectasia, status of adjacent mucosa) to establish four grades of rectal toxicity。 |
| **Notes** |  |

#### Risk of bias table

| **Bias** | **Authors' judgement** | **Support for judgement** |
| --- | --- | --- |
| Random sequence generation (selection bias) | Unclear risk | Not mentioned |
| Allocation concealment (selection bias) | Unclear risk | Not mentioned |
| Blinding of participants and personnel (performance bias) | Unclear risk | Not mentioned |
| Blinding of outcome assessment (detection bias) | Unclear risk | Not mentioned |
| Incomplete outcome data (attrition bias) | Unclear risk | Not mentioned |
| Selective reporting (reporting bias) | Unclear risk | no protocol |
| Other bias | Low risk |  |

### *Wang 2019*

| **Methods** | Randomised control trail |
| --- | --- |
| **Participants** | Patients who underwent pelvic radiotherapy |
| **Interventions** | Daily oral dietary fibre 15g |
| **Outcomes** | Bodyweight, BMI, haemoglobin, albumin, prealbumin, lymphocyte subsets |
| **Notes** |  |

#### Risk of bias table

| **Bias** | **Authors' judgement** | **Support for judgement** |
| --- | --- | --- |
| Random sequence generation (selection bias) | Unclear risk | "randomly assigned" |
| Allocation concealment (selection bias) | Unclear risk | "randomly assigned" |
| Blinding of participants and personnel (performance bias) | High risk | Not mentioned and the application of TCM may be identical |
| Blinding of outcome assessment (detection bias) | Unclear risk | Not mentioned |
| Incomplete outcome data (attrition bias) | Low risk | 7 participants dropout deal to lost of follow-up, lack of result data or no proper drug-ingestion |
| Selective reporting (reporting bias) | Unclear risk | no protocol |
| Other bias | Low risk |  |

### *Xiao 2019*

| **Methods** | Randomized control trail |
| --- | --- |
| **Participants** | Patients who received intensive modulated radiation therapy (IMRT) |
| **Interventions** | The moxibustion group gave the therapy of moxibustion on the acupuncture point of Shenque, Qihai, Guanyuan and smectite orally; observe group take smectite orally only |
| **Outcomes** | Morbidity rate  Severity of CRP |
| **Notes** |  |

#### Risk of bias table

| **Bias** | **Authors' judgement** | **Support for judgement** |
| --- | --- | --- |
| Random sequence generation (selection bias) | Unclear risk | "Randomly assigned" |
| Allocation concealment (selection bias) | Unclear risk | "Randomly assigned" |
| Blinding of participants and personnel (performance bias) | High risk | Not mentioned and the application of TCM may be identical |
| Blinding of outcome assessment (detection bias) | Unclear risk | Not mentioned |
| Incomplete outcome data (attrition bias) | Low risk | 3 dropouts, 1 deal to lost of contact and two died of "other diseases" |
| Selective reporting (reporting bias) | Unclear risk | no protocol |
| Other bias | Low risk |  |

### *Yuan 2019*

| **Methods** | Randomized control trail |
| --- | --- |
| **Participants** | Patients met include criteria: |
| **Interventions** | The treatment group was treated with Fuzheng Qingchang Yin combined with probiotics, while the control group was treated with probiotics alone for 28 days. |
| **Outcomes** | Comparing the clinical efficacy evaluation, syndrome integral evaluation, stool routine examination changes, colonoscopy changes, quality of life changes, and safety evaluation of the two groups before and after treatment |
| **Notes** |  |

#### Risk of bias table

| **Bias** | **Authors' judgement** | **Support for judgement** |
| --- | --- | --- |
| Random sequence generation (selection bias) | Low risk | Doll's clinical case randomisation table |
| Allocation concealment (selection bias) | Low risk | "According to the principle of random grouping, add the last two digits of the date of visit or hospitalizations number, and then add the last two digits of the sum to the Dolls Clinical Randomization Table (see Appendix 4). Those whose digits intersect at T are assigned to the treatment group, and those at C are assigned to the control group" |
| Blinding of participants and personnel (performance bias) | High risk | it may be differentiable between TCM drink and probiotic capsule |
| Blinding of outcome assessment (detection bias) | Unclear risk | Not mentioned |
| Incomplete outcome data (attrition bias) | Low risk | 4 exclusions "due to the deterioration of the condition during the observation period, failure to take the medicine according to the regulations, review or unauthorized change of treatment methods" |
| Selective reporting (reporting bias) | Unclear risk | no protocol |
| Other bias | Low risk |  |

## Supplementary table series S2. Characteristics of excluded studies

### *Esco, R. 2004*

| **Reason for exclusion** | Drug was not oral administrated (i.m.) |
| --- | --- |

### *Euctr, G. B. 2014*

| **Reason for exclusion** | Study completed in 2019 but report not found |
| --- | --- |

### *Kennedy 2001*

| **Reason for exclusion** | Cohort study without control |
| --- | --- |

### *Levitsky, J. 2003*

| **Reason for exclusion** | Full text not found. Same research group and research topic with [Ehrenpreis, E.D. 2005](file:///C:\\Users\\HUAWEI\\Desktop\\meta%E5%88%86%E6%9E%90\\revman%20export\\Oral%20Drugs%20for%20Chronic%20Radiation%20Proctitis.htm#STD-Ehrenpreis_x002c_-E.D.-2005) |
| --- | --- |

### *Nomoto, S. 2004*

| **Reason for exclusion** | Observational study without control |
| --- | --- |

### *Patel, P. 2009*

| **Reason for exclusion** | Observational study without control |
| --- | --- |

### *P Delia 2007*

| **Reason for exclusion** | This study probably focused on acute radiation proctitis as mentioned in introduction section. Thus not associated with our topic |
| --- | --- |

### *Venkitaraman, R. 2008*

| **Reason for exclusion** | Observational study without control |
| --- | --- |

### *Yang 2017*

| **Reason for exclusion** | Observational study without control |
| --- | --- |

### *Yao 2014*

| **Reason for exclusion** | This study actually focused on acute radiation proctitis and the drugs were administrated both orally and though enemas. |
| --- | --- |

### *Yuan 2009*

| **Reason for exclusion** | Observational study without control |
| --- | --- |

##

## Supplementary table series S3. Characteristics of ongoing studies

### *6thHospitalSunYat-senUniversity 2020*

| **Study name** | Thalidomide in the Treatment of Chronic Radiation Proctitis With Intractable Bleeding (Thal-CRP) |
| --- | --- |
| **Methods** | Prospective, Open Clinical Trial |
| **Participants** | NA |
| **Interventions** | The patients were treated with thalidomide tanken orally every night for 4 months, and the treatment period was divided into induction period and maintenance period, as follows:  Induction period:  The oral dose of thalidomide started at 50 mg, and increased to 100 mg after one week if tolerable, and maintain 100 mg for three weeks. The medication time was 1 month.  Maintenance period: The oral dose of thalidomide was 50-75mg/d. The medication time was 3 months. |
| **Outcomes** | Primary Outcome Measures: Remission rate of rectal bleeding one month after thalidomide treatment [Time Frame: the first month after thalidomide treatment] Remission of rectal bleeding was defined as the score of Subjective Objective Management Analysis system (SOMA) for retcal bleeding at least 1 grade lower and the hemoglobin level at least 10g/L higher than that before treatment. The SOMA score for hematochezia was ranged from 1 to 4, with the higher the score, the more serious.  Secondary Outcome Measures :Remission rate of rectal bleeding one month during thalidomide treatment [ Time Frame: the first month during thalidomide treatment ]Remission of rectal bleeding was defined as the score of Subjective Objective Management Analysis system (SOMA) for retcal bleeding at least 1 grade lower and the hemoglobin level at least 10g/L higher than that before treatment. The SOMA score for hematochezia was ranged from 1 to 4, with the higher the score, the more serious. Remission rate of rectal bleeding two months during thalidomide treatment [ Time Frame: the second month during thalidomide treatment] Remission of rectal bleeding was defined as the score of Subjective Objective Management Analysis system (SOMA) for retcal bleeding at least 1 grade lower and the hemoglobin level at least 10g/L higher than that before treatment. The SOMA score for hematochezia was ranged from 1 to 4, with the higher the score, the more serious. Remission rate of rectal bleeding three months during thalidomide treatment [ Time Frame: the third month during thalidomide treatment] Remission of rectal bleeding was defined as the score of Subjective Objective Management Analysis system (SOMA) for retcal bleeding at least 1 grade lower and the hemoglobin level at least 10g/L higher than that before treatment. The SOMA score for hematochezia was ranged from 1 to 4, with the higher the score, the more serious. Remission rate of rectal bleeding four months during thalidomide treatment [ Time Frame: the fourth month during thalidomide treatment] Remission of rectal bleeding was defined as the score of Subjective Objective Management Analysis system (SOMA) for retcal bleeding at least 1 grade lower and the hemoglobin level at least 10g/L higher than that before treatment. The SOMA score for hematochezia was ranged from 1 to 4, with the higher the score, the more serious. Remission rate of rectal bleeding three months after thalidomide treatment [ Time Frame: the third month after thalidomide treatment] Remission of rectal bleeding was defined as the score of Subjective Objective Management Analysis system (SOMA) for retcal bleeding at least 1 grade lower and the hemoglobin level at least 10g/L higher than that before treatment. The SOMA score for hematochezia was ranged from 1 to 4, with the higher the score, the more serious. Remission rate of rectal bleeding six months after thalidomide treatment [ Time Frame: the sixth month after thalidomide treatment ]Remission of rectal bleeding was defined as the score of Subjective Objective Management Analysis system (SOMA) for retcal bleeding at least 1 grade lower and the hemoglobin level at least 10g/L higher than that before treatment. The SOMA score for hematochezia was ranged from 1 to 4, with the higher the score, the more serious. Endoscopic score one month during thalidomide treatment [ Time Frame: the first month during thalidomide treatment] Endoscopic score was performed using Vienna rectoscopy Score (VRS). VRS was ranged 1 to 5. A higher score indicated a more severe lesion. Endoscopic score four months during thalidomide treatment [ Time Frame: the fourth month during thalidomide treatment] Endoscopic score was performed using Vienna rectoscopy Score (VRS). VRS was ranged 1 to 5. A higher score indicated a more severe lesion. Endoscopic score one month after thalidomide treatment [ Time Frame: the first month after thalidomide treatment] Endoscopic score was performed using Vienna rectoscopy Score (VRS). VRS was ranged 1 to 5. A higher score indicated a more severe lesion. Endoscopic score three months after thalidomide treatment [ Time Frame: the third month after thalidomide treatment] Endoscopic score was performed using Vienna rectoscopy Score (VRS). VRS was ranged 1 to 5. A higher score indicated a more severe lesion. Endoscopic score six months after thalidomide treatment [ Time Frame: the sixth month after thalidomide treatment] Endoscopic score was performed using Vienna rectoscopy Score (VRS). VRS was ranged 1 to 5. A higher score indicated a more severe lesion. Quality of life of the patient one month during thalidomide treatment [ Time Frame: the first month during thalidomide treatment] Quality of life of the patient was evaluated by the European Organisation for the Research and Treatment of Cancer Quality of Life Questionnaire Core 30 (EORTC QLQ-C30). The EORTC QLQ-C30 is a 30-item questionnaire composed of multi-item scales and single items that reflect the multidimensionality of the quality-of-life. It incorporated five functioning subscales (physical, role, cognitive, emotional, and social), nine symptom subscales (pain, fatigue, nausea and vomiting, appetite loss, constipation, diarrhea, dyspnea, insomnia, financial difficulties), and one global quality of life subscale, and the scores were ranged 0 to 100. Higher scores for functional subscales and global quality of life subscale indicated the better functional status and quality of life. But the higher scores for symptom subscales indicated the poorer quality of life. Quality of life of the patient two months during thalidomide treatment [ Time Frame: the second month during thalidomide treatment ]Quality of life of the patient was evaluated by the European Organisation for the Research and Treatment of Cancer Quality of Life Questionnaire Core 30 (EORTC QLQ-C30). The EORTC QLQ-C30 was a 30-item questionnaire composed of multi-item scales and single items that reflecting the multidimensionality of the quality-of-life. It incorporated five functioning subscales (physical, role, cognitive, emotional, and social), nine symptom subscales (pain, fatigue, nausea and vomiting, appetite loss, constipation, diarrhea, dyspnea, insomnia, financial difficulties), and one global quality of life subscale, and the scores were ranged 0 to 100. Higher scores for functional subscales and global quality of life subscale indicated the better functional status and quality of life. But the higher scores for symptom subscales indicated the poorer quality of life. Quality of life of the patient three months during thalidomide treatment [ Time Frame: the third month during thalidomide treatment ]Quality of life of the patient was evaluated by the European Organisation for the Research and Treatment of Cancer Quality of Life Questionnaire Core 30 (EORTC QLQ-C30). The EORTC QLQ-C30 was a 30-item questionnaire composed of multi-item scales and single items that reflecting the multidimensionality of the quality-of-life. It incorporated five functioning subscales (physical, role, cognitive, emotional, and social), nine symptom subscales (pain, fatigue, nausea and vomiting, appetite loss, constipation, diarrhea, dyspnea, insomnia, financial difficulties), and one global quality of life subscale, and the scores were ranged 0 to 100. Higher scores for functional subscales and global quality of life subscale indicated the better functional status and quality of life. But the higher scores for symptom subscales indicated the poorer quality of life. Quality of life of the patient four months during thalidomide treatment [ Time Frame: the fourth month during thalidomide treatment ]Quality of life of the patient was evaluated by the European Organisation for the Research and Treatment of Cancer Quality of Life Questionnaire Core 30 (EORTC QLQ-C30). The EORTC QLQ-C30 was a 30-item questionnaire composed of multi-item scales and single items that reflecting the multidimensionality of the quality-of-life. It incorporated five functioning subscales (physical, role, cognitive, emotional, and social), nine symptom subscales (pain, fatigue, nausea and vomiting, appetite loss, constipation, diarrhea, dyspnea, insomnia, financial difficulties), and one global quality of life subscale, and the scores were ranged 0 to 100. Higher scores for functional subscales and global quality of life subscale indicated the better functional status and quality of life. But the higher scores for symptom subscales indicated the poorer quality of life. Quality of life of the patient one month after thalidomide treatment [ Time Frame: the first month after thalidomide treatment ]Quality of life of the patient was evaluated by the European Organisation for the Research and Treatment of Cancer Quality of Life Questionnaire Core 30 (EORTC QLQ-C30). The EORTC QLQ-C30 was a 30-item questionnaire composed of multi-item scales and single items that reflecting the multidimensionality of the quality-of-life. It incorporated five functioning subscales (physical, role, cognitive, emotional, and social), nine symptom subscales (pain, fatigue, nausea and vomiting, appetite loss, constipation, diarrhea, dyspnea, insomnia, financial difficulties), and one global quality of life subscale, and the scores were ranged 0 to 100. Higher scores for functional subscales and global quality of life subscale indicated the better functional status and quality of life. But the higher scores for symptom subscales indicated the poorer quality of life. Quality of life of the patient three months after thalidomide treatment [ Time Frame: the third month after thalidomide treatment ]Quality of life of the patient was evaluated by the European Organisation for the Research and Treatment of Cancer Quality of Life Questionnaire Core 30 (EORTC QLQ-C30). The EORTC QLQ-C30 was a 30-item questionnaire composed of multi-item scales and single items that reflecting the multidimensionality of the quality-of-life. It incorporated five functioning subscales (physical, role, cognitive, emotional, and social), nine symptom subscales (pain, fatigue, nausea and vomiting, appetite loss, constipation, diarrhea, dyspnea, insomnia, financial difficulties), and one global quality of life subscale, and the scores were ranged 0 to 100. Higher scores for functional subscales and global quality of life subscale indicated the better functional status and quality of life. But the higher scores for symptom subscales indicated the poorer quality of life. Quality of life of the patient six months after thalidomide treatment [ Time Frame: the sixth month after thalidomide treatment ]Quality of life of the patient was evaluated by the European Organisation for the Research and Treatment of Cancer Quality of Life Questionnaire Core 30 (EORTC QLQ-C30). The EORTC QLQ-C30 was a 30-item questionnaire composed of multi-item scales and single items that reflecting the multidimensionality of the quality-of-life. It incorporated five functioning subscales (physical, role, cognitive, emotional, and social), nine symptom subscales (pain, fatigue, nausea and vomiting, appetite loss, constipation, diarrhea, dyspnea, insomnia, financial difficulties), and one global quality of life subscale, and the scores were ranged 0 to 100. Higher scores for functional subscales and global quality of life subscale indicated the better functional status and quality of life. But the higher scores for symptom subscales indicated the poorer quality of life. Incidence of adverse events four months during thalidomide treatment [ Time Frame: the fourth month during thalidomide treatment ]Including adverse reactions of thalidomide and other adverse events Incidence of adverse events six months after thalidomide treatment [ Time Frame: the sixth month after thalidomide treatment ]Including adverse reactions of thalidomide and other adverse events |
| **Starting date** | December 22, 2020 |
| **Contact information** |  |
| **Notes** | Not yet recruiting |

### *Gunnar 2020*

| **Study name** | Dietary Fiber During Radiotherapy - a Placebo-controlled Randomized Trial (FIDURA) |
| --- | --- |
| **Methods** | a Placebo-controlled Randomized Trial |
| **Participants** |  |
| **Interventions** | The participants are invited to eat 15 capsules per day. The capsules either contain dietary fiber from psyllium husk or placebo. |
| **Outcomes** | Primary Outcome Measures:Sign of inflammation i blood [ Time Frame: One month after the end of radiotherapy ]Concentration of c-reactive protein in plasma or serum Intensity of the urgency syndrome [ Time Frame: One year after the end of radiotherapy ]Patient-reported outcomes through a validated questionnaire. The metric for the intensity of the urgency syndrome weighs the frequency of several symptoms by factor loadings, as described in PubMed ID 28158314. Examples of the previous usage can be seen in PubMed ID 28366105 and PubMed ID 30601820. Secondary Outcome Measures :Tolerance to additional dietary fiber [ Time Frame: During radiotherapy ]Deviation from the recommended intake of 15 capsules per day Signs of inflammation i blood [ Time Frame: One month after the end of radiotherapy ]Concentration of selected markers in blood and feces. The selection of cytokines and chemokines for this outcome is work in progress 26 august 2020 and will be be given as updated information later on. In feces we will primarily use elastase and calprotectin. Intensity of the fecal-leakage syndrome, uncontrolled flatulence, excess mucus discharge and blood [ Time Frame: One year after the end of radiotherapy ]Patient-reported outcomes through a validated questionnaire. The metric for the intensity of the syndromes weighs the frequency of several symptoms by factor loadings, as described in PubMed ID 28158314. Examples of the previous usage can be seen in PubMed ID 28366105 and PubMed ID 30601820. Acute side-effects [ Time Frame: During radiotherapy, primarily during week 3 ]Distress from gastrointestinal symptoms as reported through a mobile phone application (ePROMS). Primarily we ask for defecation frequency, stool consistency (Bristol Scale), and frequency of abdominal pain. The participants may also report other side-effects, either by the mobile phone application, video link, or telephone with the study secretariat. Other Outcome Measures:Composition of microbiota [ Time Frame: During, one month and one year after radiotherapy ]Metrics obtained from freshly frozen defecated feces. The investigators plan to use next-generation sequencing. |
| **Starting date** |  |
| **Contact information** |  |
| **Notes** |  |

### *Irmgard E Kronberger 2020*

| **Study name** | Micronized Purified Flavonoid- Fraction (MPFF) in the Management of Radiation Proctitis (MiFlaPRO) |
| --- | --- |
| **Methods** | multicenter, randomised, double-blind, placebo-controlled Phase III study. |
| **Participants** | chronic radiation proctitis inducing anorectal bleedings |
| **Interventions** | 500mg film-coated tablet, oral route, tablets should be taken at meal times. 4-day course of 6 tablets daily, followed by 4 tablets over the next 3 days followed by 2 tablets daily, one at midday and one in the evening; 4-day course of 6 tablets daily, followed by 4 tablets over the next 3 days followed by 2 tablets daily |
| **Outcomes** | Primary Outcome Measures: Number of necessary intervention [ Time Frame: Day 1- Day 360] Interventions to stop acute bleeding by chronic radiation proctitis: surgical, endoscopic or proctoscopic interventions Secondary Outcome Measures: Quality of life of patients [ Time Frame: Day 0 Day 180 Day 360] Differences in quality of life of patients will be measured with evaluation of two questionnaires (EORTC QLQ C30 & PRT20). Higher scores mean a better quality of life. Blood samples [ Time Frame: Day 0-Day 360 ]Differences in serum hemoglobin, thrombocytes, coagulation parameters (quick/pt=prothrombin time) Stool [ Time Frame: Day 0-Day 360 ]Differences in calprotectin stool level Presence of histological alterations [ Time Frame: Day 0-Day 360 ]Differences in histological alterations (distortion of crypts, Inflammation of crypts, fibrosis and ectasia of vessels, cytokines) |
| **Starting date** | November 3, 2020 |
| **Contact information** |  |
| **Notes** | recruiting |

### *UCLondon Hospitals 2011*

| **Study name** | Study to Prevent Radiation Induced Damage to Bowel Using a Prebiotic Enhanced Diet. |
| --- | --- |
| **Methods** | Double-blind placebo-controlled trials |
| **Participants** | patients with prostate carcinoma or 5 weeks in patients with cervical or endometrial carcinoma who are to undergo pelvic radiotherapy with intent to cure. |
| **Interventions** | dietary supplementation with 15g/day FructoOligoSaccharide (FOS) for 7.5 weeks |
| **Outcomes** | Primary Outcome Measures :Gastrointestinal Status [ Time Frame: 5 weeks or 7.5 weeks ]To determine whether there is a difference in gastrointestinal status at 5 weeks (enumerated through the Birmingham score) in patients undergoing pelvic irradiation for gynaecological malignancy or at 7.5 weeks in patients undergoing radiotherapy for prostate malignancy given a prebiotic enhanced diet and those on placebo.  Secondary Outcome Measures :Short Term Toxicity [ Time Frame: 5 weeks or 7.5 weeks ]To determine the effects of FOS on the short-term toxicity of pelvic irradiation (in comparison to placebo). See Effects of FOS [ Time Frame: 5 or 7.5 weeks, 6 months ]To establish the effects of FOS on intestinal integrity, determined endoscopically, biochemically and histologically, after pelvic irradiation, both immediately and at 6 months follow-up Effect of FOS on Chronic Radiation Bowel Disease [ Time Frame: 5 weeks or 7.5 weeks, 3 months, 6 months ]To provide pilot data to determine whether FOS given during pelvic irradiation has an effect on the risk of clinically apparent chronic radiation bowel disease. Effect on Gut Microbiota [ Time Frame: 5 weeks or 7.5 weeks, 3 months, 6 months ]To confirm using fluorescence in-situ hybridization (FISH) the changes in the gut microbiota in patients on FOS enhanced diet in comparison with standard diet. |
| **Starting date** | 2011 Aug 11 |
| **Contact information** |  |
| **Notes** | recruiting |
